# Exploring social difference among entrepreneurs in entrepreneurial ecosystems: a systematic review and intersectional framework

**DOI:** 10.1007/s11187-026-01216-5

**Published:** 2026-05-02

**Authors:** Micaela Lois, Isabella Stingl, Heike Mayer, Susann Schäfer

**Affiliations:** 1 Institute of Geography, University of Bern, Bern, Switzerland; 2 Institute of Geography, Heidelberg University, Heidelberg, Germany

**Keywords:** Entrepreneurial ecosystem, Entrepreneurship, Intersectionality, Inclusivity, Systematic literature review, L26

## Abstract

The entrepreneurial ecosystem (EE) approach has long been criticized for assuming equal access while overlooking how structural inequalities shape entrepreneurial opportunities. This article proposes a conceptual framework for an intersectional perspective of entrepreneurial ecosystems. Such a framework is necessary because EEs that are more inclusive perform better, foster greater innovation, stronger knowledge spillovers, and show higher adaptability. Based on a review of 111 publications, we systematically examine how social categories and relations of difference shape entrepreneurial experiences and EEs. We identify three main approaches to EEs: a traditional perspective treating entrepreneurs as a homogeneous group; an actor-oriented approach to understanding difference in EEs that emphasizes individual categories of difference (most often gender), and an emerging intersectional approach, which we further develop. In sum, the field has broadened its understanding of entrepreneurial identities by addressing categories such as gender, migration, age, race/ethnicity, socio-economic background, and dis/ability. However, many studies remain focused on single axes, and only a few explore how identities are strategically mobilized. To address this gap, our conceptual framework centers on the entrepreneur as a situated actor embedded in overlapping power relations. By emphasizing lived experiences and the relational dynamics of inclusion, exclusion, and agency, it lays the groundwork for more inclusive, context-sensitive, and equitable entrepreneurial environments.

## Abstract (plain language summary)

Entrepreneurial ecosystems aren’t neutral—they reflect and reproduce social inequalities. This article proposes a conceptual framework for an intersectional perspective of entrepreneurial ecosystems to foster more inclusive entrepreneurial ecosystems (EEs). Based on 111 reviewed publications this study examine how EEs research engages with social differences. We identify three main approaches: a traditional perspective treating entrepreneurs as a homogeneous group; an actor-oriented approach to understanding difference in EEs that emphasizes individual categories of difference, and an emerging intersectional approach that considers how multiple, overlapping identities affect entrepreneurial experiences. We further develop this intersectional approach by creating a conceptual framework for intersectional analysis of EEs that centers entrepreneurs as situated actors embedded in power relations, emphasizing inclusion, exclusion, and agency. This has key implications for research and policy: adopting an intersectional lens can help build more inclusive and equitable entrepreneurial ecosystems that perform better, foster greater innovation, stronger knowledge spillovers, and show higher adaptability.

## Introduction

The concept of entrepreneurial ecosystems (EEs) has emerged in recent years as an innovative approach in research, policy, and practice for understanding and promoting place-based entrepreneurial activities (Schäfer & Mayer, 2019; Stam & Ven, 2021; Wurth et al., 2022). However, the ecosystem approach has been critiqued for its implicit assumption that all entrepreneurs have equal opportunities for participation, support, and success (Brush et al., 2019; Cowell et al., 2018; Huang et al., 2022). This rather monolithic assumption overlooks how entrepreneurs’ positioning along various axes of difference such as age, class, dis/ability, family status, gender, migration status, and/or race shapes their experiences and trajectories within EEs (Pickernell et al., 2022).

Over the past years, we have seen an emergence of studies that focus on how such categories of difference and social positioning influence entrepreneurial activities (e.g., Aman et al., 2021; Csillag et al., 2019; van Merriënboer et al., 2023*). This development marks a shift towards a more differentiated, actor-oriented perspective in the EE literature—one that responds to earlier critiques that the concept of the entrepreneur within EEs has too often been shaped by an undifferentiated image of the White, male, venture-backed entrepreneur (Garcia & Baack, 2023; Welter et al., 2017; Wurth et al., 2022). Authors have shown that ecosystems are not neutral and instead reproduce power relations and inequalities (Ozkazanc-Pan, 2022; Ozkazanc-Pan & Clark Muntean, 2021b; Vershinina, 2025). Indeed, social differences among entrepreneurs — such as gender, ethnicity, class, age, and immigration — shape how ecosystems function. They influence who participates, who is perceived as a legitimate entrepreneur, which role models are visible, how resources flow, which ventures emerge, and ultimately how inclusive and innovative an ecosystem becomes.

Recognising the immense inequalities regarding access to entrepreneurial resources, particularly for marginalized population, several EE scholars (e.g., Herbertson & Lee, 2024; Ozkazanc-Pan et al., 2021; Vershinina, 2025; Yamamura et al., 2022) and others in the broader entrepreneurship research (e.g., Abbas et al., 2019; Dy & MacNeil, 2023; Knight, 2016; Vongswasdi et al., 2025) have called for a critical and intersectional understanding of ecosystems and entrepreneurship.

Intersectional approaches—as rooted in the understanding that inequalities such as gender, race, and class do not operate in isolation but intersect to shape distinct experiences (e.g., Crenshaw, 1991; Hill Collins, 2000 [1990])—can offer valuable insights into power dynamics, unequal access to resources, and processes of inclusion and exclusion in EEs. Intersectionality highlights how various dimensions of inequality and discrimination are experienced simultaneously, thus considering the multiplicity of power relations (Bilge, 2010; Rodó-Zárate, 2023). It does not only consider social categories and identities but also the structural relations of domination that shape them (Crenshaw, 1994; Rodó-Zárate, 2024).

To gain deeper insights into this evolving field, we seek to develop a conceptual framework for an intersectional analysis of EE. To do so, we conducted a systematic review of EE studies that empirically examine how social categories and relations of difference shape entrepreneurial experiences and strategies. We focus on categories and relation of differences and their intersections, as structural inequalities often become visible through the ways identities are constructed, negotiated, and positioned within ecosystem interactions.

We find that the traditional approach to studying EEs has been complemented by a more actor-oriented approach to difference in EEs, enriching understandings of ecosystem structures, components, resource access, and entrepreneurial outcomes. Yet, we identify a lack of intersectional perspectives in the current literature. To advance this understanding, and in response to scholars calling for such a perspective, we propose a concrete intersectional framework to understand how EEs can perform better. This approach centers on entrepreneurs situated and lived experiences, emphasizing the dynamic interplay of privilege and discrimination across different components of entrepreneurial ecosystems. Understanding entrepreneurs’ experiences from an intersectional perspective can inform efforts to create more inclusive ecosystems that promote social equity and can strengthen the resilience, innovative capacity, collaborative potential, and economic performance of EEs by leveraging diverse talents and perspectives (Brush et al., 2019; Stam, 2015; Sundermeier, 2024; Welter et al., 2017).

The remainder of this paper is structured as follows: First, we outline the methodology of our systematic literature review. Next, we present our findings on the current state of research, identifying key categories and relations of difference in the EE literature. We then explore how these studies can broaden and deepen traditional understandings of EEs and entrepreneurship. Finally, we introduce our intersectional framework for analyzing difference and inequality within EEs, discussing its practical applications and potential directions for future research.

## Methodology

The dynamic development in the field of EEs studies calls for a deeper exploration of social differences and inequality among entrepreneurs. We have thus decided to not only study dominant themes related to the study of categories of difference in EE research but also examine how this body of literature can expand and challenge existing models in EEs studies. To this end, we conducted a systematic literature review (SLR), which is a standalone scientific method that synthesizes and critically evaluates the current state of research on a topic to identify gaps and areas that require further investigation (Klatt, 2023).

SLRs have gained considerable importance in management and entrepreneurship studies in recent years, reflecting the growing need for structured knowledge synthesis in an increasingly fragmented field (Kraus et al., 2023). This is particularly evident in the expanding body of research on entrepreneurial ecosystems. Existing reviews seek to consolidate the fragmented nature of EEs research and capture the dynamics of various ecosystem components (e.g., Wadichar et al., 2024; Wurth et al., 2022), yet none systematically examines how social differences and power structures shape those dynamics. Other reviews focus on specific contexts such as rural ecosystems (Calispa Aguilar, 2021) or emerging economies (Cao & Shi, 2021), highlighting the importance of context-sensitive approach. Foss et al. (2019) further show how entrepreneurship policy often individualizes inequality, focusing on “fixing” women rather than addressing gendered structures, reinforcing the need for a structural perspective in EE research. Our approach builds on and extends Huang et al.’s (2022) SLR conducted in 2019, which explores how disadvantaged entrepreneurship is conceptualized in the EE literature. Based on an analysis of 23 empirical papers, Huang et al. found that while most studies focused on women entrepreneurs, other forms of disadvantage—such as those based on age, ethnicity, disability, or geographic location—remain underrepresented.

To advance the debate about the role social differences play in EE research, we posed the following research questions: 1) Which categories and relations of difference have been studied in the field of EE research to date? 2) How have the categories and relations of difference analyzed been found to influence entrepreneurial activity, particularly regarding access to or exclusion from ecosystem resources? 3) How does the focus on categories and relations of difference inform the authors’ understanding of entrepreneurship and EEs?

**Fig. 1:**
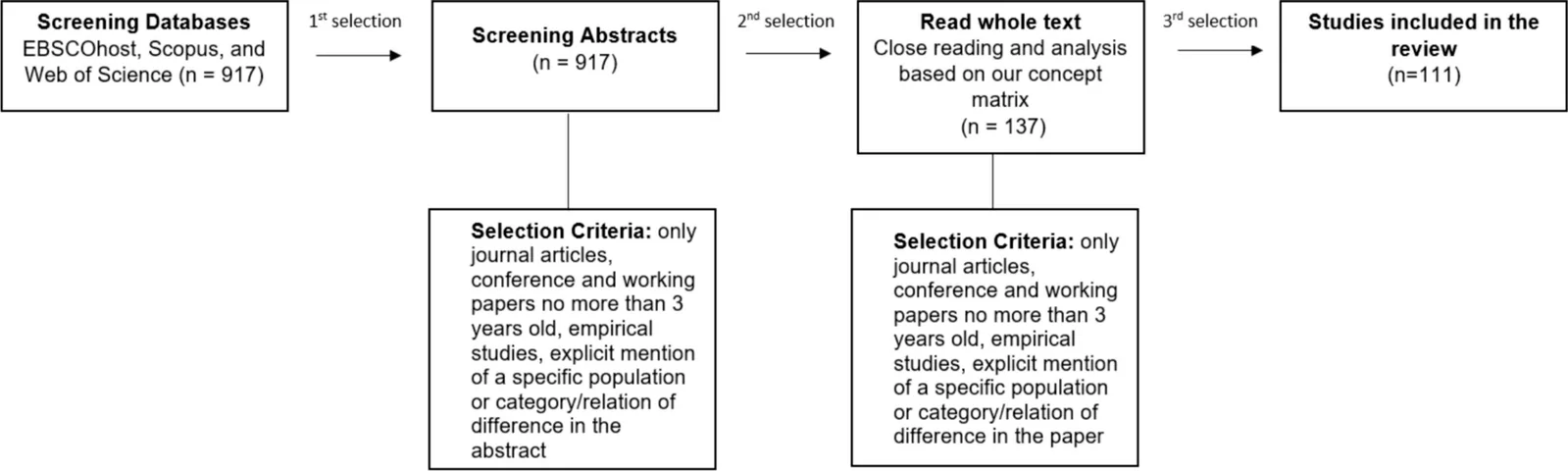
The selection process for the reviewed literature (source: authors)

We systematically explored these questions in a multi-stage procedure (see Fig. 1), starting with a literature search in the three comprehensive databases EBSCOhost, Scopus, and Web of Science. To identify relevant literature, we developed a search string (see Appendix 1) with “entrepreneurship/entrepreneurial ecosystem” as the primary keyword and then included additional keywords to capture a wide variety of categories of difference (e.g., age, gender, race), the potential expressions of these categories (e.g., young, senior, female, male, non-binary, Black, White, Indigenous), and the associated social relations of domination/subordination (e.g., ageism, patriarchy, sexism, racism). These keywords need to be mentioned in the publication’s title and/or abstract. We further restricted the search to literature published in English from 2010, when the EE framework gained traction (Malecki, 2018; Schäfer & Mayer, 2019), to March 2024. After removing duplicates that were found in more than one database, this search resulted in an initial selection of 917 publications.

We then reviewed the abstracts of these publications, applying additional inclusion and exclusion criteria: We only included journal articles, as well as both conference papers and working papers that were no more than three years old, to capture the most recent debates and dynamics in the evolving field of EEs research. We focused exclusively on empirical studies as we are looking at how social differences and intersectionality are operationalized in practice within EE research, so we excluded theoretical, conceptual, and review papers. In addition, we only selected studies that explicitly indicated in their abstracts a focus on a specific population of entrepreneurs or at least one category/relation of difference. This process resulted in a revised sample of 111 publications (see Appendix 2), which we then read and analyzed in detail.

For close reading and analysis of these publications, we developed a concept matrix (Webster & Watson, 2002) that allowed us to systematize the articles according to our research questions. This matrix is theoretically grounded in Stam and van de Ven’s (2021) ecosystem definition and elements. Within this matrix, we documented how each paper addressed these elements^1^ in relation to social differences, how categories of difference were conceptualized and how they were found to affect entrepreneurs’ experiences. The systematic literature review builds the base of this paper and was then used to examine the evolution of the field of EE research in relation to its actor orientation. We utilize the review to present an intersectional approach to the study of EEs in Section 4.

## Findings

### Charting the field: social categories of difference in entrepreneurial ecosystem studies

**Fig. 2:**
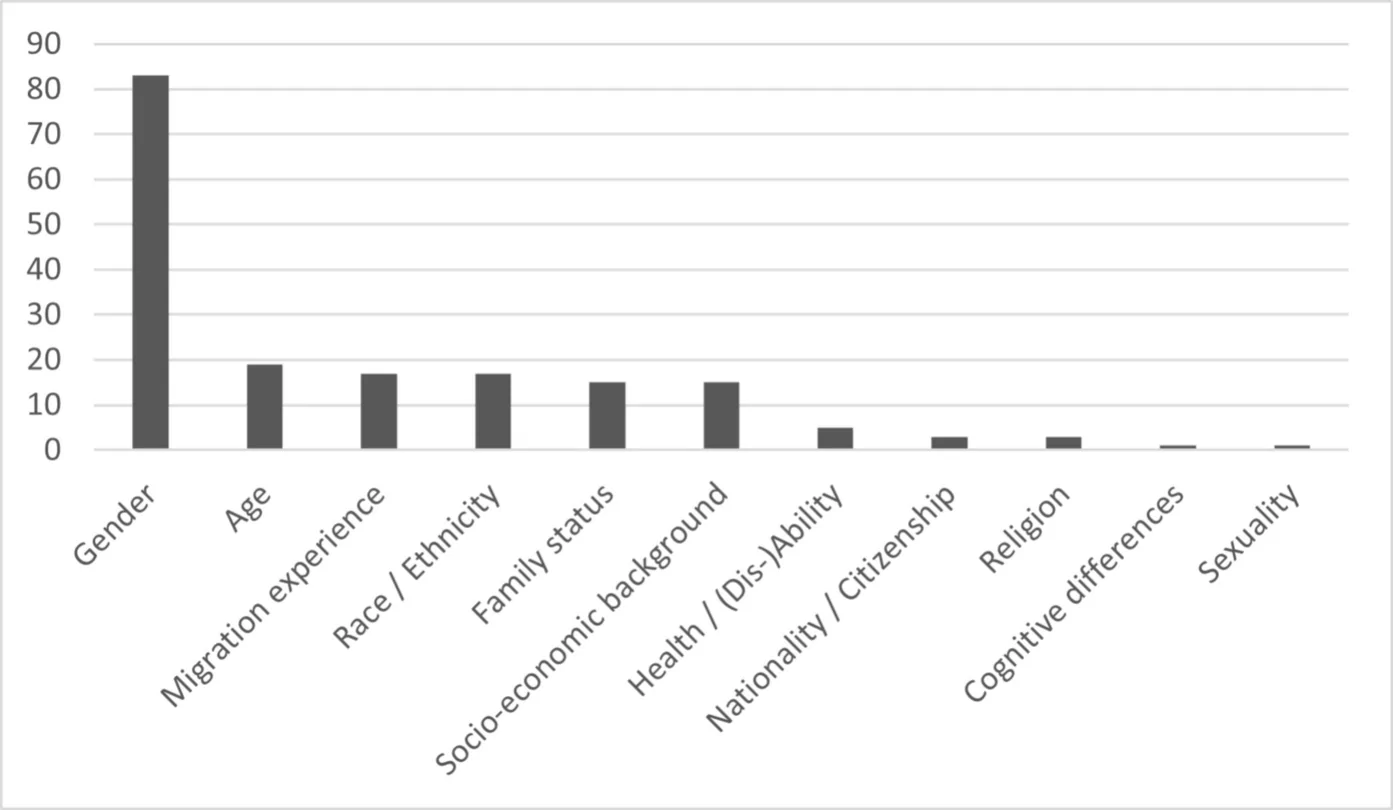
Number of articles per social category of difference (in absolute numbers) (source: authors)

Our literature review shows that, as in the 2019 review by Huang et al. (2022), gender remains the dominant category. In our analysis, over two-thirds of the reviewed articles (83) focus on gender. However, many other categories of difference are also examined (Fig. 2). Sexuality is largely absent from the literature, with just one article briefly addressing the challenges faced by LGBTQ entrepreneurs (Q. Wang & Richardson, 2021*). Cognitive differences are similarly underexplored, appearing in only one article without in depth analysis (Owalla et al., 2021*). Overall, the field of EE research lacks substantial engagement with the role of health and mental health in entrepreneurship. The same goes for other categories of difference that we searched for but did not find in the databases, such as language/accent or criminal record.

**Fig. 3:**
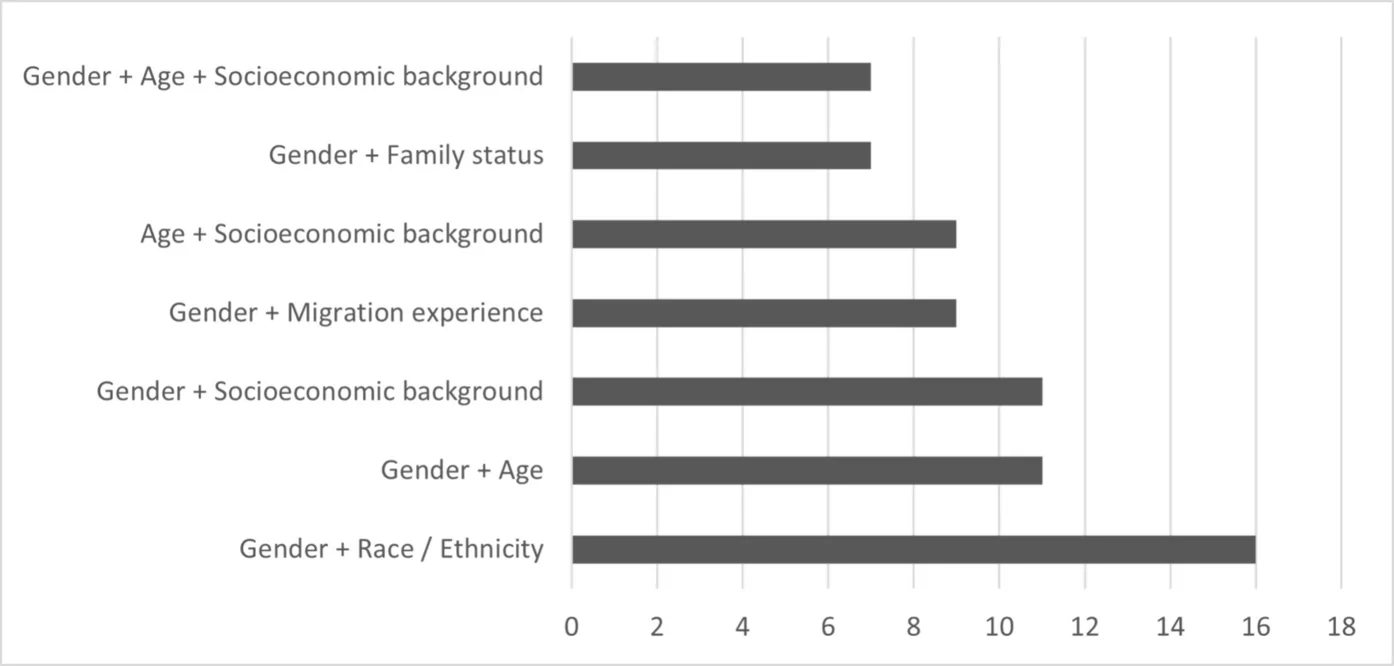
The most common combinations of social category in the reviewed papers (in absolute numbers) (source: authors)

We find that most articles (74) focus on a single category, while over 30% of the articles (37) in our sample consider multiple categories (see Fig. 3 for the most common combinations). Among the 37 multi-categorical articles, most articles analyze two categories together (22), while fewer examine three (7) or more (8) categories simultaneously.

In our SLR, several papers overlook how social categories of difference shape entrepreneurial experiences. Indeed, some studies, although including multiple social categories of difference, treat them as socio-demographic sample variables rather than incorporating them into the core analysis (Gimmon & Felzensztein, 2023; Samsami et al., 2024*). Additionally, some articles consider multiple categories but analyze them separately, focusing on each independently rather than examining how their intersection shapes specific entrepreneurial experiences.

We identified 27 papers that use an intersectional perspective exploring how entrepreneurs navigate EEs. This means that multiple social categories of difference and power relations are considered simultaneously (and not separately), and that the focus lies on how the intersection of these categories shapes the entrepreneurial experiences (e.g., Bijedić & Piper, 2019; Owalla et al., 2021*). One paper also highlights how entrepreneurs employ intersectional counter-frames that allow individuals to challenge and reinterpret dominant narratives and structures that marginalize them (Owalla et al., 2021*). Although these studies adopt an intersectional perspective, this approach could be further developed by considering the broader power structures shaping entrepreneurship experiences and the simultaneous inclusion and exclusion of entrepreneurs from specific resources within EEs.

### (Re)thinking entrepreneurial ecosystems through social differences

Identifying critical components required to interact within a given region to enable successful entrepreneurship remains a central theme in EE research. Many studies in our sample draw on established definitions of EE elements, such as institutional frameworks and resource endowments. However, when exploring entrepreneurs’ experiences and social differences with these elements, authors often emphasize the disabling functions of EEs and their components (e.g., Csillag et al., 2019*).

It is particularly the large number of studies on women entrepreneurs that demonstrate how EEs can have a disabling function. Authors like Adegbile et al., (2024*), Chauke (2022*), Freund et al., (2024*), Mehtap et al., (2017*), and Aman et al., (2021*) show how patriarchal societal structures shape the institutional frameworks of EEs, leading to gendered norms and cultures that can exclude women entrepreneurs from essential resources, such as networks, funding (Bonin et al., 2021; Lawson & Lahiri-Dutt, 2020; Rana et al., 2022*), lack of institutional support (J. Wang et al., 2019*), legal constraints (Adegbile et al., 2024*), and restrictive social norms (Kim et al., 2020; Motoyama et al., 2021; Rica, 2021*).

From this perspective, differences in EEs cannot be attributed solely to individual entrepreneurs. Instead, attention needs to also be paid to the broader systems of dominance––such as ableism, ageism, classism, racism, and patriarchy––that create and sustain uneven power relations in EEs. While they manifest in phenomena like gendered norms and cultures, the uneven power relations they (re)produce permeate and influence all interactions within the ecosystem (Ozkazanc-Pan, 2022; Ozkazanc-Pan & Clark Muntean, 2021a; Vershinina, 2025; Welter, 2011; Yamamura et al., 2022).

However, the reviewed studies do not only focus on the disabling functions of ecosystem elements and their structural embeddedness. They also emphasize those elements within ecosystems that can help to overcome barriers and exclusion (Ingole & Sohani, 2024; Khokhawala & Iyer, 2021*). For example, a significant number of studies focus on the role of universities as key hubs, especially for female, young, and migrant entrepreneurs (e.g., Amornsiripanitch et al., 2021; Civera & Meoli, 2023; Cochran, 2021; Mehtap et al., 2017; Neumeyer, 2022*). In addition, many studies draw attention to specific networks—such as informal, friendship, co-ethnic, diaspora, and indigenous networks (e.g., Duan et al., 2022; Lawson & Lahiri-Dutt, 2020; Mrabure et al., 2021; Ratten & Pellegrini, 2020; Schmutzler et al., 2021; Tamtik, 2020*)—as well as the crucial role of the family, which can enable entrepreneurship despite exclusion from more traditional resources. The reviewed studies suggest that families play a multifaceted role in fostering entrepreneurship, providing emotional and moral support while facilitating access to critical resources such as land, workspaces, and capital (Bugawa & Aljuwaisri, 2019; Darcy et al., 2023; Gimmon & Felzensztein, 2023; Luis-Rico et al., 2020; Welsh et al., 2023*).

Digital business models are another resource, Duan et al., (2022*) find that cross-border e-commerce platforms significantly contribute to the success of migrant and transnational entrepreneurs as they facilitate access to multiple ecosystems. The importance of digital spaces is not only emphasized in relation to migration. Ditta-Apichai et al., (2024*) demonstrate how online social media platforms support women’s micro-entrepreneurship in Thailand’s tourism sector. The authors describe how these platforms cultivate digital spaces for knowledge-sharing, mentorship, and emotional support, offering women entrepreneurs a vital alternative ecosystem in an environment shaped by patriarchal norms and limited resources (see also Abdelwahid & Kaoud, 2022*).

Regarding entrepreneurial purpose and outcome, several studies highlight women’s entrepreneurship as a path to economic empowerment, independence, self-realization, and social recognition at the individual level (e.g., Khan & Sharpe, 2016; Mehtap et al., 2017*). Similarly, for entrepreneurs living with disabilities, independence is identified as an important goal and potential outcome of entrepreneurship (Darcy et al., 2023*). In the context of indigenous entrepreneurship, studies emphasize that entrepreneurial activities play a crucial role in preserving indigenous culture and ensuring the survival and continuity of these communities (Mika et al., 2022; Mrabure et al., 2021; Tamtik, 2020*). Wang and Richardson (2021*) describe how artist entrepreneurs in the USA are motivated by the structural disadvantages they face due to gender, class, race, and ethnicity, which inspire them to establish social enterprises aimed at tackling inequality and supporting marginalized groups. On a broader societal level, Rica (2021*), Ditta-Apichai et al., (2024*), and Khan and Sharpe (2016*) discuss the potential of women’s entrepreneurial endeavors, regardless of the specific business model, to act as trailblazers by challenging societal expectations and transforming gender roles in post-communist and patriarchal societies.

While social differences among entrepreneurs are increasingly discussed in the EE literature, heterogeneity across other ecosystem actors (e.g., investors, policymakers, support organizations) remains largely under-studied. Only a few reviewed articles such as Prencipe et al., (2023*) analyze diversity in university spin-off boards and other authors (Kumar & Das, 2019; Mehtap et al., 2017; Rica, 2021; Simmons et al., 2024*) demonstrate how socio-cultural norms, gendered political environment and patriarchal structures influence entrepreneurial processes. Greater actor diversity within ecosystems would foster more inclusive spaces, institutions, and policies, and consequently greater diversity among successful entrepreneurs.

In summary, the studies we reviewed illustrate that by focusing on social differences they can challenge and expand conventional understandings of EEs. Embedded in uneven power relations, EEs can hinder or complicate entrepreneurial activity for certain groups. Yet the reviewed studies pay limited attention to the positive ways in which social identities can shape entrepreneurial activities or how entrepreneurs leverage their identities as assets. These differences matter because they influence who participates in entrepreneurship, how resources flow, which ventures are created, and how inclusive and innovative the ecosystem becomes. At the same time, these studies broaden the conceptual scope of EEs by highlighting spatialities and components that are often overlooked—such as local neighborhoods, the intimate sphere of the home, digital business platforms, or social media. While neighborhoods may offer communal support, the domestic sphere emerges as a central site where women, in particular, often run their businesses and receive crucial familial or spousal support. In doing so, the reviewed literature deepens our understanding of the geographical and relational contexts in which entrepreneurship unfolds (Stam & Welter, 2020).

Moreover, the reviewed studies respond to long-standing calls for more diversity-sensitive research (Mazzoni et al., 2025; Welter et al., 2017; Wurth et al., 2022), illustrating that entrepreneurship takes diverse forms with the potential to empower individuals, families, and communities, and, crucially, that ecosystems perform more effectively when intersectional social categories are considered, contributing to broader societal transformation.

## Future directions: an intersectional research agenda for studying difference in entrepreneurial ecosystems

The current debate on EEs can broadly be divided into two approaches: a traditional and an actor-oriented approach to understanding differences in EEs. Based on the concept of intersectionality—which emphasizes a simultaneous examination of social categories of difference in terms of how they intersect and interact to produce specific experiences (Crenshaw, 1991; Hill Collins, 2000 [1990])—we propose a third, intersectional approach. As we have demonstrated, intersectional perspectives are already present within existing approaches to EEs. In this section, however, we aim to further develop these perspectives into a more systematic framework—one that allows for an understanding not only of entrepreneurial experiences at the individual level, but also of the broader outcomes and evolutionary trajectories of EEs. In Table 1, we seek to identify the types of approaches and the categories in which they follow different assumptions and goals.

**Table 1:** Towards an intersectional approach to studying EEs

	Traditional approach to EEs	Actor-oriented understanding of difference in EEs	Intersectional approach to difference in EEs
EE Literature	Literature does not differentiate the entrepreneur Social categories are not explicit	Literature started to differentiate, but mostly based on one category (e.g., female entrepreneur) Multiple categories are emerging, but analyzed separately	Social categories are analyzed interrelatedly Entrepreneurs’ experiences are analyzed as situated in space and time
Under-standing of EE	Neutral sites for entrepreneurial activities	Sites of exclusion and discrimination resulting from structural embeddedness	Sites of inclusion and exclusion embedded in intersecting power relations
Actor Orientation	Undifferentiated understanding of entrepreneurs and other EEs actors Idealized archetype of White, male, capital-backed entrepreneur	Growing focus on specific groups of entrepreneurs Predominantly mono-categorical, especially centered on female entrepreneurs	Understanding of entrepreneurs and all other EE actors as positioned along multiple intersecting categories of difference
Ecosystem Components	One-size-fits-all model with a focus on enabling function of components Economic-centric perspective	Exposure of disabling function of established components Identification of alternative, individually enabling components	Enabling/disabling function of components dependent on intersectional positioning and contextual power dynamics
Resource Access	Assumption of equal access for all entrepreneurs Unequal access is result of fair competition	Exposure of unequal distribution and structural constraints	Dynamic understanding of access and constraints shaped by context-specific, intersecting power relations
Entrepreneurial Outcomes	New value creation as outcome of productive entrepreneurship Productive entrepreneurship is influenced by systemic and framework conditions	New value creation as outcome of productive entrepreneurship Productive entrepreneurship is influenced by systemic and framework conditions, which in turn are conditioned by one or multiple social categories	New value creation can be an outcome of intersectional experiences Experience of systemic and framework conditions is influenced by intersecting social categories Strategic entrepreneurial response to barriers and exclusions
Methodology	Focus primarily on successful case study regions or high-profile entrepreneurs Traditional methods (quantitative and/or qualitative)	Social categories used to sample specific entrepreneurs Traditional methods (quantitative and/or qualitative)	Methodology takes multiple axis of identity into account Inclusive research designs & methods that center lived experiences

The early phases of scholarship of EEs were marked by an approach that viewed the system as rather monolithic. This traditional approach to EEs is based on the assumption that all entrepreneurs are equal. They have the same opportunities or at least it is assumed that opportunities are the result of fair competition in the ecosystem. The academic studies here do not differentiate between entrepreneurs, and social categories of difference are not explicitly considered. EEs are understood as neutral spaces of economic activity (Malecki, 2018; Ozkazanc-Pan & Clark Muntean, 2021a). Entrepreneurs are seen as a rather homogeneous group, with the image of the White, male, well-funded entrepreneur dominating (Garcia & Baack, 2023; Welter et al., 2017). This led to a “one-size-fits-all” model that was business-centric and assumed that all entrepreneurs just need the right training of skills to be able to be ‘successful’ (Brown & Mason, 2017; Brush et al., 2019). Productive entrepreneurship is defined exclusively as the creation of new value (jobs, innovation, growth, etc.), while research focused primarily on successful regions or prominent entrepreneurs (Mazzoni et al., 2025; Stam, 2015; Wurth et al., 2022), using traditional qualitative or quantitative methods. This approach is criticized because of its simplification of social processes and the focus on entrepreneurial output and success (e.g., number of unicorns, amount of VC).

Over the past years, there has been a move towards a more differentiated perspective as was shown in this literature review. Research began to differentiate, but mostly along individual categories, especially gender. It was increasingly recognized – as shown in this literature review—that entrepreneurial ecosystems are not neutral but can reproduce exclusion and discrimination through structural embedding. As a result, the consideration of specific groups becomes more important, although often only a single category of difference is examined. At the same time, it becomes clear that existing components of the ecosystem can not only be beneficial but also inhibiting. Unequal access to resources becomes apparent, which is attributed to structural barriers (Motoyama et al., 2021*) and unequal distribution. Entrepreneurial results are no longer viewed solely as individual value creation, but as a product of systemic and structural framework conditions (Stam, 2015; Stam & van de Ven, 2021). Research methods begin to use social categories particularly to study specific groups of entrepreneurs but often remain anchored in traditional quantitative or qualitative methods.

The analysis of the literature underlines, however, that there is still an untapped potential to integrate an intersectional perspective in studies of EEs. While earlier approaches paint an undifferentiated picture of entrepreneurs, current research shows that social categories of difference play an increasingly important role. Building on these insights, and in line with scholars such as Herbertson and Lee (2024), Ozkazanc-Pan (2022; and Ozkazanc-Pan & Clark Muntean, 2021b), Vershinina (2025), and Yamamura et al. (2022), we argue that a more systematic integration of intersectionality into EE research is beneficial for understanding ecosystem outcomes and evolutionary trajectories.

Through an intersectional lens, EEs can be understood as spaces in which inclusion and exclusion are shaped by overlapping power relations. Entrepreneurs and other ecosystem actors are analyzed not as homogenous groups, but as individuals positioned along multiple, intersecting categories of difference. Methodologically, this perspective supports approaches that attend to multiple axes of identity, consider institutional and structural exclusion mechanisms (Ozkazanc-Pan, 2022; Vershinina, 2025), apply inclusive research designs, and center the lived and situated experiences (Rodó-Zárate, 2023; Valentine, 2007; Yuval-Davis, 2015) of entrepreneurs and other ecosystem actors. An intersectional perspective does not merely add categories of difference but fundamentally reshapes how EEs are conceptualized — positioning power relations as a central analytical dimension (Ozkazanc-Pan, 2022).

**Fig. 4:**
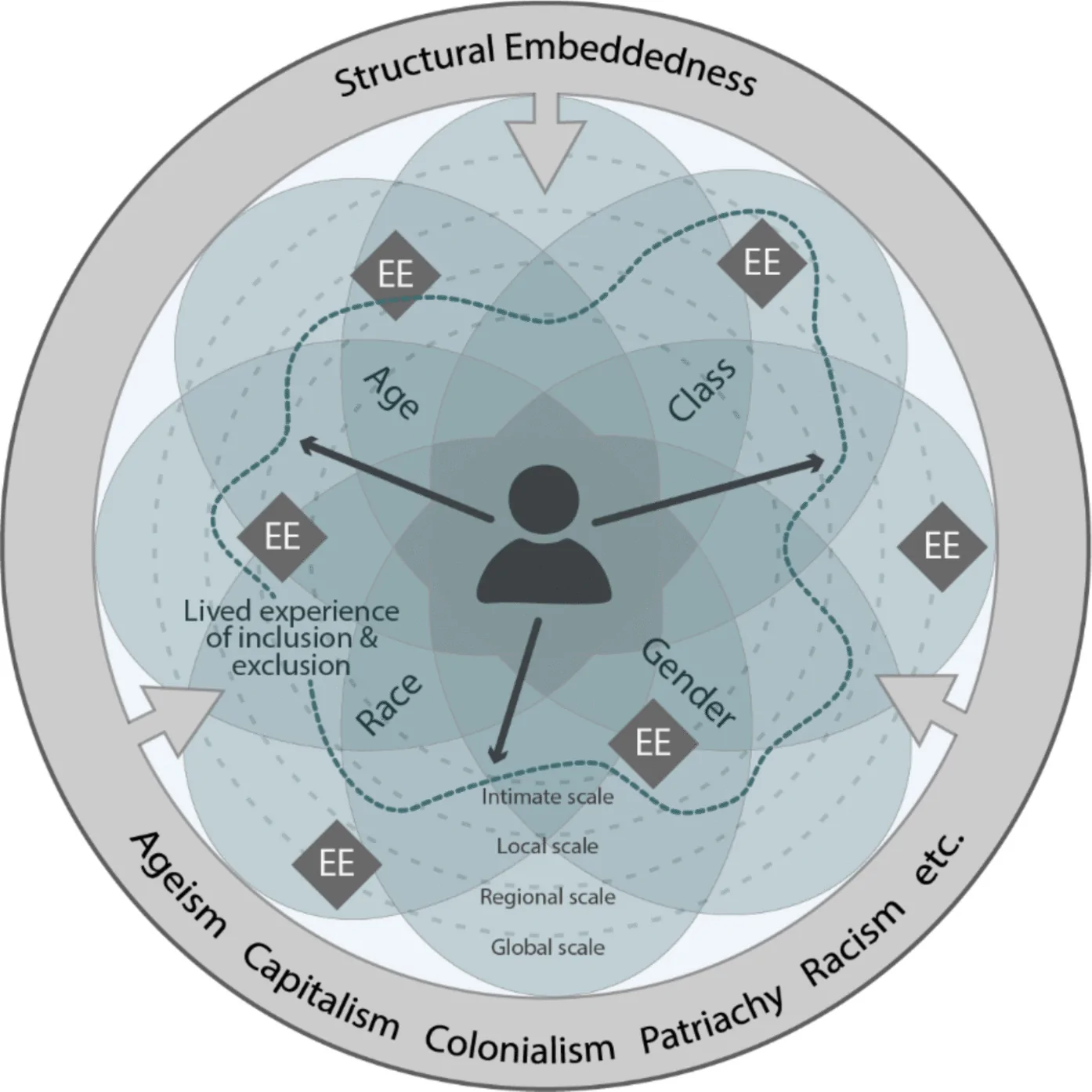
An intersectional framework for research on entrepreneurial ecosystems (source: authors)

Figure 4 illustrates a conceptual framework for studying EEs from an intersectional perspective: At the center is the entrepreneur, positioned along intersecting categories such as gender, age, race, or class, which shape their lived experiences within the EE. As our literature review shows, these experiences rarely involve full inclusion or total exclusion. Rather, entrepreneurs navigate differentiated experiences of inclusion in and exclusion from individual EE components, which can be situated across multiple spatial scales, from the intimate to the global. Whether entrepreneurs are able to access these components—and whether they enable or inhibit entrepreneurial activity—depends on how broader power structures such as patriarchy, racism, colonialism, capitalism, and ageism manifest in specific contexts (Dy & MacNeil, 2023).

With Fig. 4, we show not only the influence of the embeddedness of entrepreneurial ecosystems within intersecting power relations but also offer a framework that foregrounds entrepreneurial agency (Stam & Welter, 2020). The outward-facing arrows from the entrepreneur symbolize this reciprocal relationship—entrepreneurs are shaped by their environments but also act upon them. While much of the literature highlights constraints tied to social positioning, we argue that more attention should be paid to how entrepreneurs strategically deploy aspects of their intersectional identities (cf. Nash, 2008)—not only to navigate barriers, but also to seize position-specific opportunities for productive entrepreneurship. One rare example in the reviewed literature is van Merriënboer et al., (2023*), who show how tech entrepreneurs in the Netherlands initially downplay identity markers such as race or gender to avoid discrimination but later reclaim them to build authenticity and legitimacy—what we argue may also alter the broader structural relations over time. An intersectional perspective on such reciprocal dynamics thus also contributes to an evolutionary view of entrepreneurial ecosystems (Mack & Mayer, 2016), where outcomes are shaped by how entrepreneurs respond to and reconfigure the structures they are embedded in.

## Conclusion

This article systematically examines the dynamic debate on how social differences among entrepreneurs are conceptualized and studied in EE research. Drawing on a review of 111 publications, we identify three main approaches: a traditional approach, which assumes equal access and treats entrepreneurs as a homogeneous group; an actor-oriented approach, which highlights differentiated experiences—often along a single axis such as gender—and to which most reviewed studies can be attributed; and an emerging intersectional approach, which we seek to further develop.

In sum, the field has expanded its understanding of entrepreneurial identities by considering categories such as gender, migration experience, age, race/ethnicity, socio-economic background, and dis/ability. Social differences in ecosystems matter because they influence participation in entrepreneurship, resource distribution, the types of ventures that emerge, and the overall inclusiveness of the ecosystem. However, many categories remain underexplored or entirely overlooked, such as health, religious affiliation, citizenship, sexuality, languages, or criminal records. In addition, our review shows that heterogeneity across other ecosystem actors (e.g., investors, policymakers, support organizations) remains largely under-studied. Future research would benefit from focusing on these dimensions.

While most studies focus on single categories, some begin to adopt intersectional perspectives that consider their mutual shaping. In addition, recent research broadens the scope of EE studies by emphasizing entrepreneurship as a potential site of empowerment, social change, and community resilience—extending beyond economic output. It also brings attention to overlooked spaces such as the home, the family, and digital platforms. However, few studies examine how entrepreneurs strategically mobilize aspects of their identity to navigate or reshape ecosystem structures.

As with any systematic literature review, this study has certain limitations. First, while our analysis provides a comprehensive overview of the existing research on difference in EEs, it is inherently characterized by the selection criteria, databases, and search terms we used, which may have excluded relevant studies outside these parameters. Literature reviews are inherently affected by publication bias, as they predominantly include studies published in peer-reviewed journals and may overlook unpublished or emerging research that could provide additional insights or expand the scope of existing debates. Furthermore, our review is limited to articles published in English, potentially overlooking valuable insights from research conducted in other languages and contexts. Moreover, as we focus on EE studies, we do not take into account the broader debate on entrepreneurship that has already provided more comprehensive insights into intersectionality and social inequalities (e.g., Abbas et al., 2019; Dy & MacNeil, 2023; Knight, 2016; Vongswasdi et al., 2025; Yamamura et al., 2022). The EE literature could therefore greatly benefit from a more intensive engagement with these debates.

Despite these limitations, our review provides a structured foundation for future research and highlights both the advances and blind spots in the current literature. To move beyond additive or essentialist notions of difference, we propose a conceptual framework for an intersectional analysis of EEs. This framework centers on the entrepreneur as a situated actor embedded in overlapping power relations—such as racism, patriarchy, capitalism, and coloniality—and emphasizes the dynamic and context-specific interplay between inclusion, exclusion, and entrepreneurial agency. It enables a more nuanced understanding of how ecosystem structures are both shaped by and responsive to the positionalities of actors within them. We offer this framework as a basis for future empirical and theoretical work that seeks to better understand and ultimately transform EEs into more inclusive, equitable, and innovative spaces.

An intersectional understanding of ecosystems also has implications for policies. They should be more differentiated and responsive to the diverse social positions of entrepreneurs and to power relations that shape them. Policies are not neutral, as institutions tend to reflect and reinforce existing structures of inequality (Vershinina, 2025). A critical starting point is therefore to develop more systematic data collection practices that capture social differences among entrepreneurs — including gender, race, migration status, and other axes of marginalization — to make structural inequalities visible. Such data would enable policymakers and ecosystem actors to move beyond one-size-fits-all approaches and design targeted, evidence-based measures that foster inclusive entrepreneurial opportunities and support structures for all. Indeed, entrepreneurial ecosystems that are inclusive, diverse, and socially connected would perform better, fostering greater innovation, stronger knowledge spillovers, and higher adaptability.

## Data Availability

Not applicable.

## Appendix 1: search string

TITLE-ABS ("entrepreneur* ecosystem*") AND TITLE-ABS (age OR young OR senior OR agism OR gender OR women OR woman OR female OR man OR men OR male OR non-binary OR sexism OR patriarch* OR sexual* OR lgbt* OR lesbian* OR gay* OR queer OR transgender OR bisexual OR heteronorm* OR heterosexism OR homophobia OR biphobia OR transphobia OR family OR famili* OR married OR parent* OR single-parent OR spous* OR child* OR race OR racial* OR racis* OR ethnic* OR color OR colour OR black OR white OR indigenous OR caste OR immigra* OR migra* OR refugee* OR language OR native OR non-native OR nationality OR citizen* OR religi* OR disab* OR mental OR neurodivergent OR impair* OR autis* OR adhd OR depress* OR ableism OR "education* background" OR "education* level" OR "level of education" OR "academic background" OR marginal* OR minorit* OR criminal OR convict* OR incarcerat* OR rehablitat* OR poverty OR poor OR low-income OR class OR rural OR "developing countr*" OR "developed countr*" OR "emerging econom*" OR "global north" OR "global south" OR industrialized OR industrialised OR peripher* OR underrepresented OR under-represented OR vulnerab* OR exclu* OR inequalit* OR unequal OR discriminat* OR stigma*).

## Appendix 2: list of reviewed articles

Abbas, A., Byrne, J., Galloway, L., & Jackman, L. (2019). Gender, intersecting identities, and entrepreneurship research: An introduction to a special section on intersectionality. *International Journal of Entrepreneurial Behavior & Research*, *25*(8), 1703–1705. https://doi.org/10.1108/IJEBR-11-2019-823

Abdelwahid, M., & Kaoud, H. (2022). Factors Affecting the Success of Women Entrepreneurs in Egypt. *International Journal of Organizational Leadership*, *11*(4), 444–461. (rayyan-1247469880). https://doi.org/10.33844/ijol.2022.60344

Adegbile, A. S., Ogundana, O. M., & Adesola, S. (2024). Gender-based policies and women’s entrepreneurship: An fsQCA analysis of sub-Saharan African countries. *International Journal of Entrepreneurial Behaviour and Research*. (rayyan-1247469464). https://doi.org/10.1108/IJEBR-04-2023-0394

Aman, R., Ahokangas, P., & Zhang, X. (2021). Migrant Women Entrepreneurs and Entrepreneurial Ecosystems during an External Shock: A Case Study from the Healthcare Sector in Kazakhstan. *Asian Business and Management*, *20*(4), 518–548. ecn. https://doi.org/10.1057/s41291-021-00151-5

Amornsiripanitch, N., Gompers, P. A., Hu, G., & Vasudevan, K. (2021). *Getting Schooled: The Role of Universities in Attracting Immigrant Entrepreneurs*. (rayyan-1247470019). https://search.ebscohost.com/login.aspx?direct=true&db=ecn&AN=1906439&site=ehost-live

Bijedić, T., & Piper, A. (2019). Different strokes for different folks: The job satisfaction of the self-employed and the intersection of gender and migration background. *International Journal of Gender and Entrepreneurship*, *11*(3), 227–247. (rayyan-1247469720). https://doi.org/10.1108/IJGE-01-2019-0021

Bilge, S. (2010). De l’analogie à l’articulation: Théoriser la différenciation sociale et l’inégalité complexe. *L’Homme la Societe*, *176–177*(2), 43–64.

Bonin, S., Singh, W., Suresh, V., Rashed, T., Uppaal, K., Nair, R., & Bhavani, R. R. (2021). A priority action roadmap for women’s economic empowerment (PARWEE) amid COVID-19: A co-creation approach. *International Journal of Gender and Entrepreneurship*, *13*(2), 142–161. (rayyan-1247469626). https://doi.org/10.1108/IJGE-09-2020-0148

Brown, R., & Mason, C. (2017). Looking inside the spiky bits: A critical review and conceptualisation of entrepreneurial ecosystems. *Small Business Economics*, *49*(1), 11–30. https://doi.org/10.1007/s11187-017-9865-7

Brush, C., Edelman, L. F., Manolova, T., & Welter, F. (2019). A gendered look at entrepreneurship ecosystems. *Small Business Economics*, *53*(2), 393–408. https://doi.org/10.1007/s11187-018-9992-9

Bugawa, A., & Aljuwaisri, S. M. (2019). Internal and external factors on women’s entrepreneurship performance in the state of Kuwait. *Arab Gulf Journal of Scientific Research*, *37*(3), 33–53. (rayyan-1277120685).

Calispa Aguilar, E. (2021). Rural Entrepreneurial Ecosystems: A Systematic Literature Review for Advancing Conceptualisation. *Entrepreneurial Business and Economics Review*, *9*(4), 101–114. ecn.

Cao, Z., & Shi, X. (2021). A systematic literature review of entrepreneurial ecosystems in advanced and emerging economies. *Small Business Economics*, *57*(1), 75–110. bsu. https://doi.org/10.1007/s11187-020-00326-y

Chauke, T. A. (2022). Gender and entrepreneurship: Towards a transformative agenda for young women. *Youth Voice Journal*, 3–23. (rayyan-1277120794).

Civera, A., & Meoli, M. (2023). Empowering female entrepreneurs through university affiliation: Evidence from Italian academic spinoffs. *Small Business Economics*, *61*(3), 1337–1355. (rayyan-1247469861). https://doi.org/10.1007/s11187-022-00729-z

Cochran, S. L. (2021). Creating a space for women entrepreneurs. *Rutgers Business Review*, *6*(2), 161–167. (rayyan-1247469634).

Cowell, M., Lyon-Hill, S., & Tate, S. (2018). It takes all kinds: Understanding diverse entrepreneurial ecosystems. *Journal of Enterprising Communities*, *12*(2), 178–198. Scopus. https://doi.org/10.1108/JEC-08-2017-0064

Crenshaw, K. (1991). Mapping the Margins: Intersectionality, Identity Politics, and Violence against Women of Color. *Stanford Law Review*, *43*(6), 1241–1299. https://doi.org/10.2307/1229039

Crenshaw, K. (1994). Mapping the Margins: Intersectionality, Identity Politics, and Violence Against Women of Color. In *The Public Nature of Private Violence*. Routledge.

Csillag, S., Gyori, Z., & Svastics, C. (2019). Long and winding road?: Barriers and supporting factors as perceived by entrepreneurs with disabilities. *Journal of Enterprising Communities*, *13*(1), 42–63. (rayyan-1247469775). https://doi.org/10.1108/JEC-11-2018-0097

Darcy, S., Collins, J., & Stronach, M. (2023). Entrepreneurs with disability: Australian insights through a social ecology lens. *Small Enterprise Research*, *30*(1), 24–48. (rayyan-1247469907). https://doi.org/10.1080/13215906.2022.2092888

Ditta-Apichai, M., Gretzel, U., & Kattiyapornpong, U. (2024). Platform empowerment: Facebook’s role in facilitating female micro-entrepreneurship in tourism. *Journal of Sustainable Tourism*, *32*(3), 540–559. (rayyan-1247469876). https://doi.org/10.1080/09669582.2023.2215479

Duan, C., Kotey, B., & Sandhu, K. (2022). The Effects of Cross-Border E-Commerce Platforms on Transnational Digital Entrepreneurship: Case Studies in the Chinese Immigrant Community. *Journal of Global Information Management*, *30*(2). (rayyan-1247469826). https://doi.org/10.4018/JGIM.20220301.oa2

Dy, A. M., & MacNeil, H. (2023). “Doing inequality, doing intersectionality”: Intersectionality as threshold concept for studying inequalities in entrepreneurial activity. *International Journal of Entrepreneurial Behavior & Research*, *ahead-of-print*(ahead-of-print). (world). https://doi.org/10.1108/IJEBR-12-2022-1113

Foss, L., Henry, C., Ahl, H., & Mikalsen, G. H. (2019). Women’s entrepreneurship policy research: A 30-year review of the evidence. *Small Business Economics*, *53*(2), 409–429. bsu. https://doi.org/10.1007/s11187-018-9993-8

Freund, D., Ramírez García, I., Boluk, K. A., Canut-Cascalló, M., & López-Planas, M. (2024). Exploring the gendered tourism entrepreneurial ecosystem in Barcelona and responses required: A feminist ethic of care. *Journal of Sustainable Tourism*, *32*(3), 637–655. (rayyan-1247469869). https://doi.org/10.1080/09669582.2023.2207780

Garcia, R., & Baack, D. W. (2023). The Invisible Racialized Minority Entrepreneur: Using White Solipsism to Explain the White Space. *Journal of Business Ethics*, *188*(3), 397–418. https://doi.org/10.1007/s10551-022-05308-6

Gimmon, E., & Felzensztein, C. (2023). The emergence of family entrepreneurship in the transition economy of Cuba. *International Journal of Emerging Markets*, *18*(9), 2239–2258. (rayyan-1247469437). https://doi.org/10.1108/IJOEM-09-2020-1099

Herbertson, M., & Lee, N. (2024). Inclusive Entrepreneurial Ecosystems. In R. Huggins, P. Thompson, F. Kitagawa, C. Theodoraki, & D. Prokop (Eds.), *Entrepreneurial Ecosystems in Cities and Regions: Emergence, Evolution, and Future* (p. 0). Oxford University Press. https://doi.org/10.1093/oso/9780192866264.003.0012

Hill Collins, P. (2000). *Black feminist thought: Knowledge, Consciousness, and the Politics of Empowerment* (2nd edition). Routledge.

Huang, S., Pickernell, D., Battisti, M., Dann, Z., & Ekinsmyth, C. (2022). Disadvantaged Entrepreneurship and the Entrepreneurial Ecosystem: A Critical Literature Review and Introduction. In *Disadvantaged Entrepreneurship and the Entrepreneurial Ecosystem* (Vol. 14, pp. 1–8). Emerald Publishing Limited. (world). https://doi.org/10.1108/S2040-724620220000014001

Ingole, N., & Sohani, S. S. (2024). Concurrent Interplay of Entrepreneurs – Intermediaries – Stakeholders in Emerging Economies at Different Phases of Entrepreneurial Process. *Journal of Social Entrepreneurship*, 1–21. (rayyan-64020663). https://doi.org/10.1080/19420676.2024.2303458

Khan, R. M., & Sharpe, N. (2016). Intrinsic Subtleties of Saudi Arabian Female Startups. *Journal of Women’s Entrepreneurship and Education*, (3), 35–65. (rayyan-1277120797).

Khokhawala, S., & Iyer, R. (2021). Entrepreneurial ecosystems: Spanning the institutional gaps in emerging economies via incubator networks. *Journal of the International Council for Small Business*, *2*(3), 177–202. (rayyan-64020596). https://doi.org/10.1080/26437015.2021.1881930

Kim, M., Abdullah, S. C., Bich, N. T., & Boey, I. (2020). Female entrepreneurship in the ICT sector: Success factors and challenges. *Asian Women*, *36*(2), 43–72. (rayyan-1277120678). https://doi.org/10.14431/aw.2020.6.36.2.43

Klatt, D. F. (2023). *Business Systematic Literature Reviews*.

Knight, M. (2016). Race-ing, Classing and Gendering Racialized Women’s Participation in Entrepreneurship. *Gender, Work & Organization*, *23*(3), 310–327. https://doi.org/10.1111/gwao.12060

Kraus, S., Mahto,Raj V., & and Walsh, S. T. (2023). The importance of literature reviews in small business and entrepreneurship research. *Journal of Small Business Management*, *61*(3), 1095–1106. https://doi.org/10.1080/00472778.2021.1955128

Kumar, S., & Das, S. (2019). An extended model of theory of planned behaviour: Entrepreneurial intention, regional institutional infrastructure and perceived gender discrimination in India. *Journal of Entrepreneurship in Emerging Economies*, *11*(3), 369–391. (rayyan-1247469713). https://doi.org/10.1108/JEEE-09-2018-0089

Lawson, L., & Lahiri-Dutt, K. (2020). Women sapphire traders in Madagascar: Challenges and opportunities for empowerment. *Extractive Industries and Society*, *7*(2), 405–411. (rayyan-1247469651). https://doi.org/10.1016/j.exis.2019.07.009

Luis-Rico, M.-I., Escolar-Llamazares, M.-C., de la Torre-Cruz, T., Herrero, Á., Jiménez, A., Val, P. A., Palmero-Cámara, C., & Jiménez-Eguizábal, A. (2020). The association of parental interest in entrepreneurship with the entrepreneurial interest of Spanish youth. *International Journal of Environmental Research and Public Health*, *17*(13), 1–16. (rayyan-1247469648). https://doi.org/10.3390/ijerph17134744

Mack, E., & Mayer, H. (2016). The evolutionary dynamics of entrepreneurial ecosystems. *Urban Studies*, *53*(10), 2118–2133. https://doi.org/10.1177/0042098015586547

Malecki, E. J. (2018). Entrepreneurship and entrepreneurial ecosystems. *Geography Compass*, *12*(3), e12359. https://doi.org/10.1111/gec3.12359

Mazzoni, L., Naudé, W., & Innocenti, N. (2025). The myth of entrepreneurship as a tool: Reorienting business venturing as a goal in itself in a post-growth society. *Journal of Business Venturing Insights*, *23*, e00512. https://doi.org/10.1016/j.jbvi.2024.e00512

Mehtap, S., Pellegrini, M. M., Caputo, A., & Welsh, D. H. B. (2017). Entrepreneurial intentions of young women in the Arab world: Socio-cultural and educational barriers. *International Journal of Entrepreneurial Behaviour and Research*, *23*(6), 880–902. (rayyan-1247469779). https://doi.org/10.1108/IJEBR-07-2017-0214

Mika, J. P., Felzensztein, C., Tretiakov, A., & Macpherson, W. G. (2022). Indigenous entrepreneurial ecosystems: A comparison of Mapuche entrepreneurship in Chile and Māori entrepreneurship in Aotearoa New Zealand. *Journal of Management and Organization*. (rayyan-1247469622). https://doi.org/10.1017/jmo.2022.15

Motoyama, Y., Clark Muntean, S., Knowlton, K., & Ozkazanc-Pan, B. (2021). Causes of the Gender Divide within Entrepreneurship Ecosystems. *Local Economy*, *36*(3), 187–204. (rayyan-1247469975).

Mrabure, R. H. O., Ruwhiu, D., & Gray, B. (2021). Indigenous entrepreneurial orientation: A Māori perspective. *Journal of Management & Organization*, *27*(1), 62–86. (rayyan-1247469932). https://doi.org/10.1017/jmo.2018.43

Nash, J. C. (2008). Re-Thinking Intersectionality. *Feminist Review*, *89*(1), 1–15. https://doi.org/10.1057/fr.2008.4

Neumeyer, X. (2022). Inclusive High-Growth Entrepreneurial Ecosystems: Fostering Female Entrepreneurs’ Participation in Incubator and Accelerator Programs. *IEEE Transactions on Engineering Management*, *69*(4), 1728–1737. (rayyan-1247469494). https://doi.org/10.1109/TEM.2020.2979879

Owalla, B., Vorley, T., Coogan, T., Smith, H. L., & Wing, K. (2021). Absent or overlooked? Promoting diversity among entrepreneurs with public support needs. *International Journal of Entrepreneurial Venturing*, *13*(3), 231–261. (rayyan-1247469572). https://doi.org/10.1504/IJEV.2021.116841

Ozkazanc-Pan, B. (2022). Rethinking Social Capital: Entrepreneurial Ecosystems as Contested Communities. In R. N. Eberhart, M. Lounsbury, & H. E. Aldrich (Eds.), *Entrepreneurialism and Society: Consequences and Meanings* (Vol. 82, p. 0). Emerald Publishing Limited. https://doi.org/10.1108/S0733-558X20220000082004

Ozkazanc-Pan, B., & Clark Muntean, S. (2021a). *Entrepreneurial Ecosystems: A Gender Perspective*. Cambridge University Press. https://doi.org/10.1017/9781009023641

Ozkazanc-Pan, B., & Clark Muntean, S. (2021b). Intersectional Analysis: Gender, Race, and Immigrant Status in Entrepreneurial Ecosystems. In *Entrepreneurial Ecosystems: A Gender Perspective* (pp. 184–218). Cambridge University Press.

Ozkazanc-Pan, B., Osorio, A. E., Dutta, D. K., Gupta, V. K., Javadian, G., & Guo, G. C. (2021). Modern Classics in Entrepreneurship Studies: Building the Future of the Field. In *Modern Classics in Entrepreneurship Stud.: Building the Future of the Field* (p. 235). Springer International Publishing. Scopus. https://doi.org/10.1007/978-3-030-61029-6

Pickernell, D. G., Battisti, M., Dann, Z., & Ekinsmyth, C. (2022). *Disadvantaged Entrepreneurship and the Entrepreneurial Ecosystem*. Emerald Publishing Limited. http://ebookcentral.proquest.com/lib/unibern/detail.action?docID=6846511

Prencipe, A., Boffa, D., Papa, A., Corsi, C., & Mueller, J. (2023). Unmasking intellectual capital from gender and nationality diversity on university spin-offs’ boards: A study on non-linear effects upon firm innovation. *Journal of Intellectual Capital*, *24*(1), 257–282. (rayyan-1247469449). https://doi.org/10.1108/JIC-08-2021-0207

Rana, S., Kiminami, L., & Furuzawa, S. (2022). Role of entrepreneurship in regional development in the haor region of Bangladesh: A trajectory equifinality model analysis of local entrepreneurs. *Asia-Pacific Journal of Regional Science*, *6*(3), 931–960. (rayyan-1247469520). https://doi.org/10.1007/s41685-022-00241-y

Ratten, V., & Pellegrini, M. M. (2020). Female transnational entrepreneurship and smart specialization policy. *Journal of Small Business & Entrepreneurship*, *32*(6), 545–566. (rayyan-1247469981). https://doi.org/10.1080/08276331.2019.1616257

Rica, R. (2021). Women entrepreneurship framework in Albania. *Journal of Economy and Business*, 156–172. (rayyan-64020677). https://doi.org/10.46458/27121097.2021.PI.156

Rodó-Zárate, M. (2023). Intersectionality and the Spatiality of Emotions in Feminist Research. *The Professional Geographer*, *75*(4), 676–681. https://doi.org/10.1080/00330124.2022.2075406

Rodó-Zárate, M. (2024). Geographical Dimensions of Intersectionality. In B. Akkan, J. Hahmann, C. Hunner-Kreisel, & M. Kuhn (Eds.), *Overlapping Inequalities in the Welfare State: Strengths and Challenges of Intersectionality Framework* (pp. 21–30). Springer International Publishing. https://doi.org/10.1007/978-3-031-52227-7_2

Samsami, M., Peña-Legazkue, I., & Barakat, S. (2024). The role of entrepreneurial ecosystems in reducing the gender gap of entrepreneurial intention and exit rates. *Eur. J. Int. Manage.*, *22*(4), 576–591. Scopus. https://doi.org/10.1504/EJIM.2024.137352

Schäfer, S., & Mayer, H. (2019). Entrepreneurial ecosystems: Founding figures and research frontiers in economic geography. *Zeitschrift Für Wirtschaftsgeographie*, *63*(2–4), 55–63. https://doi.org/10.1515/zfw-2019-0008

Schmutzler, J., Andonova, V., & Perez-Lopez, J. (2021). The Role of Diaspora in Opportunity-Driven Entrepreneurial Ecosystems: A Mixed-Methods Study of Balkan Economies. *International Entrepreneurship and Management Journal*, *17*(2), 693–729. (rayyan-1247469984). https://doi.org/10.1007/s11365-020-00708-4

Simmons, S. A., Lee, C. K., Young, S., Shelton, L., & Massey, M. (2024). Effects of gender equality and social costs of failure on early-stage entrepreneurship activity. *New England Journal of Entrepreneurship*. (rayyan-1247469452). https://doi.org/10.1108/NEJE-01-2023-0003

Stam, E. (2015). Entrepreneurial Ecosystems and Regional Policy: A Sympathetic Critique. *European Planning Studies*, *23*(9), 1759–1769. https://doi.org/10.1080/09654313.2015.1061484

Stam, E., & van de Ven, A. (2021). Entrepreneurial ecosystem elements. *Small Business Economics*, *56*(2), 809–832. https://doi.org/10.1007/s11187-019-00270-6

Stam, E., & Welter, F. (2020). Geographical Contexts of Entrepreneurship: Spaces, Places and Entrepreneurial Agency. In *The Psychology of Entrepreneurship*. Routledge.

Sundermeier, J. (2024). ‘It just seems that they don’t act like men’: The influence of gender role stereotypes on women’s entrepreneurial innovation activities. *Journal of Business Research*, *185*, 114902. https://doi.org/10.1016/j.jbusres.2024.114902

Tamtik, M. (2020). Informing canadian innovation policy through a decolonizing lens on Indigenous entrepreneurship and innovation. *Canadian Journal of Higher Education*, *50*(3), 63–78. (rayyan-1277120761).

Valentine, G. (2007). Theorizing and Researching Intersectionality: A Challenge for Feminist Geography*. *The Professional Geographer*, *59*(1), 10–21. https://doi.org/10.1111/j.1467-9272.2007.00587.x

van Merriënboer, M., Verver, M., & Radu-Lefebvre, M. (2023). “Really being yourself”? Racial minority entrepreneurs navigating othering and authenticity through identity work. *International Journal of Entrepreneurial Behaviour and Research*. (rayyan-1247469446). https://doi.org/10.1108/IJEBR-01-2023-0037

Vershinina, N. (2025). Gender, diversity, and entrepreneurship: Insights from feminist theories. In *Research Handbook on Entrepreneurship and Diversity* (pp. 31–45). Edward Elgar Publishing. https://www.elgaronline.com/edcollchap/book/9781803924151/chapter2.xml

Vongswasdi, P., de Groote, J., Heinrich, J., & Ladge, J. (2025). Beyond the prototype: Unpacking the intersectional identity and image work of female minority founders in a startup context. *Journal of Applied Psychology*, *110*(5), 697–722. https://doi.org/10.1037/apl0001234

Wadichar, R. K., Manusmare, P., & Burghate, M. A. (2024). Entrepreneurial Ecosystem: A Systematic Literature Review. *Vision*, *28*(2), 143–156. https://doi.org/10.1177/09722629221093866

Wang, J., Li, Y., & Long, D. (2019). Gender gap in entrepreneurial growth ambition: The role of culturally contingent perceptions of the institutional environment in China. *International Journal of Entrepreneurial Behaviour and Research*, *25*(6), 1283–1307. (rayyan-1247469706). https://doi.org/10.1108/IJEBR-04-2018-0248

Wang, Q., & Richardson, L. (2021). Fostering Art and Cultural Entrepreneurship in Underserved Communities: A Case of Newark, NJ. *Journal of Planning Education and Research*. (rayyan-1277120679). https://doi.org/10.1177/0739456X211035836

Webster, J., & Watson, R. T. (2002). Analyzing the Past to Prepare for the Future: Writing a Literature Review. *MIS Quarterly*, *26*(2), xiii–xxiii.

Welsh, D. H. B., Kaciak, E., Fadairo, M., Doshi, V., & Lanchimba, C. (2023). How to erase gender differences in entrepreneurial success? Look at the ecosystem. *Journal of Business Research*, *154*. (rayyan-1247469505). https://doi.org/10.1016/j.jbusres.2022.113320

Welter, F. (2011). Contextualizing Entrepreneurship—Conceptual Challenges and Ways Forward. *Entrepreneurship Theory and Practice*, *35*(1), 165–184. https://doi.org/10.1111/j.1540-6520.2010.00427.x

Welter, F., Baker, T., Audretsch, D. B., & Gartner, W. B. (2017). Everyday Entrepreneurship—A Call for Entrepreneurship Research to Embrace Entrepreneurial Diversity. *Entrepreneurship Theory and Practice*, *41*(3), 311–321. https://doi.org/10.1111/etap.12258

Wurth, B., Stam, E., & Spigel, B. (2022). Toward an Entrepreneurial Ecosystem Research Program. *Entrepreneurship Theory and Practice*, *46*(3), 729–778. https://doi.org/10.1177/1042258721998948

Yamamura, S., Lassalle, P., & Shaw, E. (2022). Intersecting where? The multi-scalar contextual embeddedness of intersectional entrepreneurs. *Entrepreneurship & Regional Development*, *34*(9–10), 828–851. https://doi.org/10.1080/08985626.2022.2120086

Yuval-Davis, N. (2015). Situated Intersectionality and Social Inequality. *Raisons Politiques*, *58*(2), 91–100.

## Footnotes

1 Stam and van de Ven’s (2021) elements are: Formal institutions, Culture, Networks, Physical infrastructure, Finance, Leadership, Talent, Knowledge, Demand and Intermediate services.
